# Simultaneous Assessment of White Matter Changes in Microstructure and Connectedness in the Blind Brain

**DOI:** 10.1155/2016/6029241

**Published:** 2016-01-05

**Authors:** Nina Linde Reislev, Tim Bjørn Dyrby, Hartwig Roman Siebner, Ron Kupers, Maurice Ptito

**Affiliations:** ^1^Danish Research Centre for Magnetic Resonance, Centre for Functional and Diagnostic Imaging and Research, Copenhagen University Hospital Hvidovre, 2650 Hvidovre, Denmark; ^2^BRAINlab and Neuropsychiatry Laboratory, Department of Neuroscience and Pharmacology, Faculty of Health and Medical Sciences, University of Copenhagen, 2200 Copenhagen, Denmark; ^3^Department of Neurology, Copenhagen University Hospital Bispebjerg, 2400 Copenhagen, Denmark; ^4^University of Montreal, School of Optometry, Montréal, QC, Canada H3C 3J7

## Abstract

Magnetic resonance imaging (MRI) of the human brain has provided converging evidence that visual deprivation induces regional changes in white matter (WM) microstructure. It remains unclear how these changes modify network connections between brain regions. Here we used diffusion-weighted MRI to relate differences in microstructure and structural connectedness of WM in individuals with congenital or late-onset blindness relative to normally sighted controls. Diffusion tensor imaging (DTI) provided voxel-specific microstructural features of the tissue, while anatomical connectivity mapping (ACM) assessed the connectedness of each voxel with the rest of the brain. ACM yielded reduced anatomical connectivity in the corpus callosum in individuals with congenital but not late-onset blindness. ACM did not identify any brain region where blindness resulted in increased anatomical connectivity. DTI revealed widespread microstructural differences as indexed by a reduced regional fractional anisotropy (FA). Blind individuals showed lower FA in the primary visual and the ventral visual processing stream relative to sighted controls regardless of the blindness onset. The results show that visual deprivation shapes WM microstructure and anatomical connectivity, but these changes appear to be spatially dissociated as changes emerge in different WM tracts. They also indicate that regional differences in anatomical connectivity depend on the onset of blindness.

## 1. Introduction

Structural and functional alterations in the brain related to visual deprivation have been widely studied in both animals and humans to unravel the underlying plastic mechanisms [1–3]. These lines of research have identified cross-modal compensatory plasticity and impaired brain maturation as two concepts directly related to lack of visual input [4–6]. Cross-modal plasticity refers to the ability of the blind brain to functionally recruit the visual cortex to process a variety of nonvisual tasks such as audition and language [7–10], tactile discrimination and spatial navigation [11–14], and even olfaction [15] (for review see Kupers and Ptito [6, 16]). This raises the question of whether the functional recruitment of the visual cortex during nonvisual tasks involves plastic changes in the anatomical connections. This question can be studied noninvasively with functional and structural MRI relating changes in functional connectivity to changes in structural connectivity. Yet connectivity analyses provide no information about the regional structural changes driving the connectivity differences, for instance, local microstructural changes in specific tract-systems. Linking such regional microstructural changes in white matter (WM) tracts to the altered patterns of brain connectivity can hold important information on the rewiring of the blind brain.

Volumetry based on T1-weighted structural MRI has previously been used to show regional WM and grey matter (GM) decreases in congenital and early blindness [17, 18], mainly in the primary visual pathway and the visual cortex. While volumetry indicates a deprivation-induced regional brain atrophy, diffusion MRI provides complementary information about local microstructural features in specific WM pathways [19]. Diffusion tensor imaging (DTI) employs a diffusion tensor model to characterise regional tissue properties based on well-established measures such as mean and radial diffusivity and fractional anisotropy [20–22]. Several DTI studies identified a decrease in FA in the primary visual pathway in both the early and late blind [23–27]. These regional voxel-based measures provide important information about local microstructural alterations in the blind brain but do not reflect connectivity properties of the brain. Shu and Li [28, 29] studied network properties in congenitally and early blind individuals, based on graph theory and diffusion tensor tractography. They reported a decreased degree of connectivity in occipital and inferior frontal areas, while regions involved in motor and somatosensory function (precentral gyrus, supplementary motor area) and memory (hippocampus) showed an increased degree of connectivity.

Only a few studies have related changes in regional brain structure to changes in brain connectivity. For instance, Qin et al. [30] found that congenitally blind individuals showed increased thickness of the primary visual cortex, but not the secondary visual areas, and related this local structural change to decreased resting-state functional MRI (rs-fMRI) in the early visual areas. The local properties within the WM pathways leading to the observed differences of the cortical areas in the blind brain remain to be clarified. The aim of our study was to examine the structural differences that underlie the functional plastic adaptations described by others [7, 31–33]. Brain imaging studies of the blind brain should not only establish a link between structure and function, but should also examine how the various methods relate to each other and which information is given by which measure. This gap of knowledge motivated this study in which we simultaneously assessed anatomical connectivity and microstructure in the blind brain from the same diffusion MRI dataset. Microstructural properties of the tissue were assessed based on voxel-wise DTI indices, while anatomical connectivity was assessed with anatomical connectivity mapping (ACM) [34–36] using whole-brain probabilistic tractography in congenitally and late blind participants. ACM probes how much each voxel in the WM is connected with all other WM voxels in the brain and thus can detect local alterations in whole-brain anatomical connectivity in the WM.

## 2. Materials and Methods

### 2.1. Subjects

Forty-eight subjects were included in the study, as previously used in Reislev et al. [37]. The subjects were subdivided into 12 congenitally blind (CB) participants (mean age 42 ± 13 years), 15 late blind (LB) participants (mean age 52 ± 15 years; mean onset of blindness 16.6 ± 8.9 years), and 15 normally sighted (NS) controls (mean age 46 ± 13 years) matched for gender, age, education, and handedness. The inclusion criteria of blind subjects were constrained to blindness caused by pathology of peripheral origin. The congenitally blind subjects were classified as such based on self-reports of medical history and aetiology of the subjects. Since several (9 of 12) of the CB subjects suffered from blindness due to retinopathy of prematurity, an extra control group of six subjects born 6–12 weeks prematurely (PT) (mean age 28 ± 6 years) but with normal vision was included to control for the effect of prematurity. The ethics committee for the city of Copenhagen and Frederiksberg (Denmark) approved the experimental procedures (ethics protocol number (KF) 01 328 723).

### 2.2. MRI Acquisition

Diffusion-weighted images (DWIs) of the whole brain were acquired with a 32-channel head coil on a 3.0 Tesla Siemens Verio MRI system (Siemens, Erlangen, Germany). We used a twice-refocused spin-echo sequence [38] to acquire DWI at 2.3^3^ mm^3^ isotropic resolution in 61 noncollinear directions (*b*-value of 1500 s/mm^2^) and 10 non-diffusion-weighted images (*b* = 0 s/mm^2^), as previously described in [37]. A set of reversed phase encoded *b* = 0 images was acquired for preprocessing purposes. Additionally, two T1-weighted MPRAGE image volumes were acquired (TE = 2.32 ms, TR = 1900 ms, flip angle = 9, and isotropic 0.9^3^ mm^3^ voxels).

### 2.3. Data Processing and Analyses

#### 2.3.1. Preprocessing

The data were similarly processed as described by Reislev et al. [37] and briefly listed in the following. Geometric distortions related to susceptibility artefacts were minimised in the DWIs with the application of a voxel displacement map (VDM) estimated from the two reversed phase encoded images [39, 40]. Combined correction for motion and eddy currents was obtained by applying a full affine transformation of the DWIs to the mean of the* b*0-images, using normalised mutual information (SPM8, Wellcome Trust Centre for Neuroimaging, UCL, London, UK). The images were finally resliced to native DWI resolution using 3rd-order B-spline interpolation. The 61 noncollinear directions were reoriented according to the rotation of the applied transformations [41]. The T1-weighted image volumes were intensity corrected for gradient nonlinearities of the scanner [42].

#### 2.3.2. Microstructural Indices with DTI

The diffusion tensor model [20] was fitted to the preprocessed DWI dataset using nonlinear model estimation [43]. DTI indices, that is, fractional anisotropy (FA), radial diffusivity (RD), axial diffusivity (AD), and mean diffusivity (MD), were calculated using Camino (http://cmic.cs.ucl.ac.uk/camino/) [44].

#### 2.3.3. Anatomical Connectivity Mapping (ACM)

ACM summarises whole-brain probabilistic tractography information into a scalar map reflecting the relative connectivity of each voxel within the whole-brain network [34, 36]. The ACM is generated by performing whole-brain tractography, where all voxels within a brain mask are used as seeds. Hereafter, counting the number of streamlines that project through each voxel within the brain mask then generates a voxel-wise reflection of the connectedness with the rest of the brain. Hence, ACM generates a voxel-wise connectedness map which has the capability to capture the accumulation of global effects represented within pathway systems of the brain, as opposed to FA that represents voxel-wise local effects. For instance, we recently showed that ACM offers increased sensitivity and provides additional information on specific WM alterations in multiple sclerosis as compared to FA maps [36].

#### 2.3.4. Individual Brain Mask

In each individual subject, a brain mask including the whole GM and WM of the brain was used as seed and target mask for the tractography in native space. The brain mask was obtained by performing a cortical reconstruction process of the two averaged T1-weighted images within each subject (FreeSurfer v. 5.3.0, http://surfer.nmr.mgh.harvard.edu/).

#### 2.3.5. Probabilistic Tractography in Native Space

We used the probabilistic tractography method as implemented in FSL [45, 46], based on the Bayesian modelling of crossing fibres in each voxel. Default settings were applied for most tracking parameters (2000 steps per streamline, step length = 0.5, curvature threshold = 0.2). Based on a priori analysis on the stability of the ACM as a function of how many streamlines to initiate from each voxel, we chose to use 500 streamlines for the whole-brain tractography per voxel. Due to the nature of ACM, where every voxel in the brain mask is used as seed, fewer numbers of streamlines are needed per voxel to ensure statistically robust results compared with tractography using a single seed ROI [36, 47].

#### 2.3.6. Group-Based Analysis

For group-based comparisons individual ACM, FA, RD, AD, and MD maps were spatially normalised to the MNI standard space by defining a warp field between the individual FA map and the FMRIB58_FA_2 mm atlas in standard space [40]. The warp field was obtained using FSL's linear and nonlinear registration tools flirt and fnirt [48–50]. Finally, the ACM and DTI indices maps in standard space were smoothed with a 4 mm^3^ full-width half-maximum Gaussian filter, as in Lyksborg et al. [36].

### 2.4. Group-Based Statistics

A general linear model (GLM) was set up for voxel-wise comparison of the ACM and DTI indices between groups. Because exploration of the residuals by histogram inspection and the Lilliefors test [51] revealed nonnormality, a nonparametric approach was used (FSL randomise, http://fsl.fmrib.ox.ac.uk/fsl/fslwiki/Randomise), including group as the main factor, and age, gender, and number of voxels in the brain mask in native space as covariates. The permutation testing was performed with 5000 permutations and threshold-free cluster enhancement (TFCE) applied [52]. FSL randomise allows inference using the setup of a standard general linear model [53] and thus an initial *F*-test examined for group differences in the ACM and DTI indices FA, RD, AD, and MD at *p* < 0.05, family-wise error (FWE) corrected. This was followed by post hoc one-tailed two-sample *t*-tests to test for specific whole-brain differences between NS, CB, LB, and PT individuals. Using tract-specific small volume correction (SVC), we further investigated specific pathways involved in visual processing for altered local structural connectivity and microstructure. The SVC was based on the JHU-ICBM-DTI-81 [54] atlas available in FSL. The pathways investigated included the primary visual pathway (the posterior thalamic radiation including the optic radiation), the ventral (inferior longitudinal fasciculus and inferior frontooccipital fasciculus) and dorsal (superior longitudinal fasciculus) stream and the splenium, midbody, and genu of corpus callosum, and the corticospinal tract. Values were considered significant at the FWE level of *p* < 0.05.

The ACM is generated from whole-brain probabilistic tractography and therefore variation in intracranial tissue volume (ICV) or brain size, that is, different number of voxels between seeded voxels, may bias group analysis [34, 36]. We have previously reported atrophy of the primary visual pathway related to blindness [18] and therefore investigated if global brain atrophy via the ICV had a linear correlation between the different groups biasing the ACM analysis.

## 3. Results

### 3.1. Altered Anatomical Connectivity and Microstructure in the Blind Brain

#### 3.1.1. Differences in Anatomical Connectivity between CB and NS Individuals

**Figure 1:**
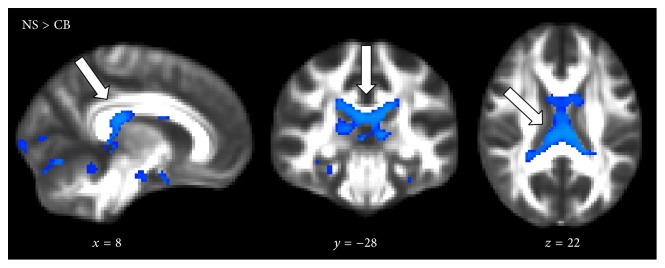
Statistical parametric maps based on whole-brain anatomical connectivity mapping (ACM) showing voxels where anatomical connectivity is significantly decreased in CB individuals relative to NS (blue voxels): NS > CB individuals (*p* < 0.05). Alterations are shown to be located mainly in the tract-system of corpus callosum (white arrows). Only voxels surviving the statistical threshold are shown, and *p* values are based on the threshold-free cluster enhancement and are family-wise error corrected. MNI-coordinates are reported in mm for *x*-, *y*-, and *z*-views. Background image is the FMRIB58_FA template.

Figure 1 shows the TFCE-based FWE corrected *t*-statistical values for whole-brain group differences in anatomical connectivity, assessed with ACM. Decreased regional ACM was found only in the CB group compared with the NS group (*p* < 0.05). This decrease was located primarily in the corpus callosum (white arrow, Figure 1). A tendency of decreased ACM was also found in the optic radiations. No significant differences were found in the whole-brain ACM between NS and LB group, but LB group differed from CB group (*p* < 0.05), suggesting that only CB individuals are affected in terms of brain connectivity. Also, ACM did not reveal significant differences in anatomical connectivity between the PT and NS individuals. In line with the observed difference between CB and NS group, CB individuals also showed decreased ACM when compared to PT individuals (*p* < 0.05). This indicates that congenital blindness and not prematurity is the cause of the reported differences.

#### 3.1.2. Differences in DTI Microstructural Features between CB, LB, and NS Individuals

**Figure 2:**
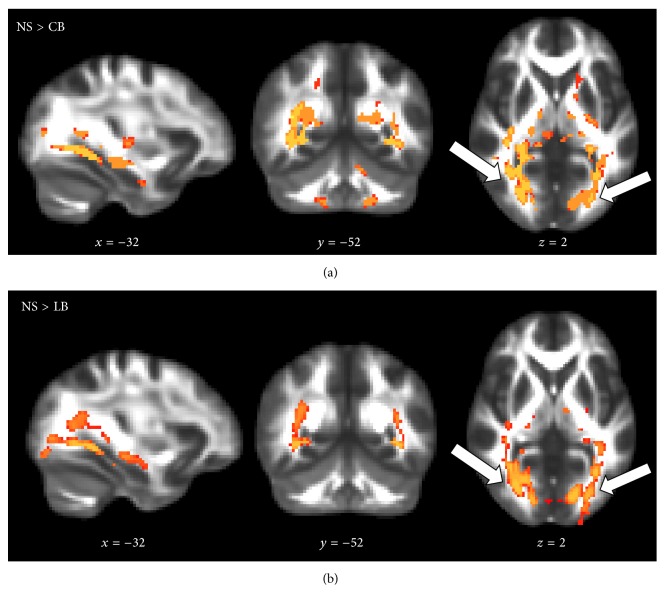
Statistical parametric FA maps showing a relative decrease in regional fractional anisotropy (FA) in CB and LB individuals relative to NS (red-yellow voxels): NS > CB individuals (*p* < 0.001, (a)) and NS > LB individuals (*p* < 0.001, (b)). The FA differences are seen in the primary visual pathway indicated by the white arrows and the ventral streams. *p* values and background image defined as in Figure 1.

**Figure 3:**
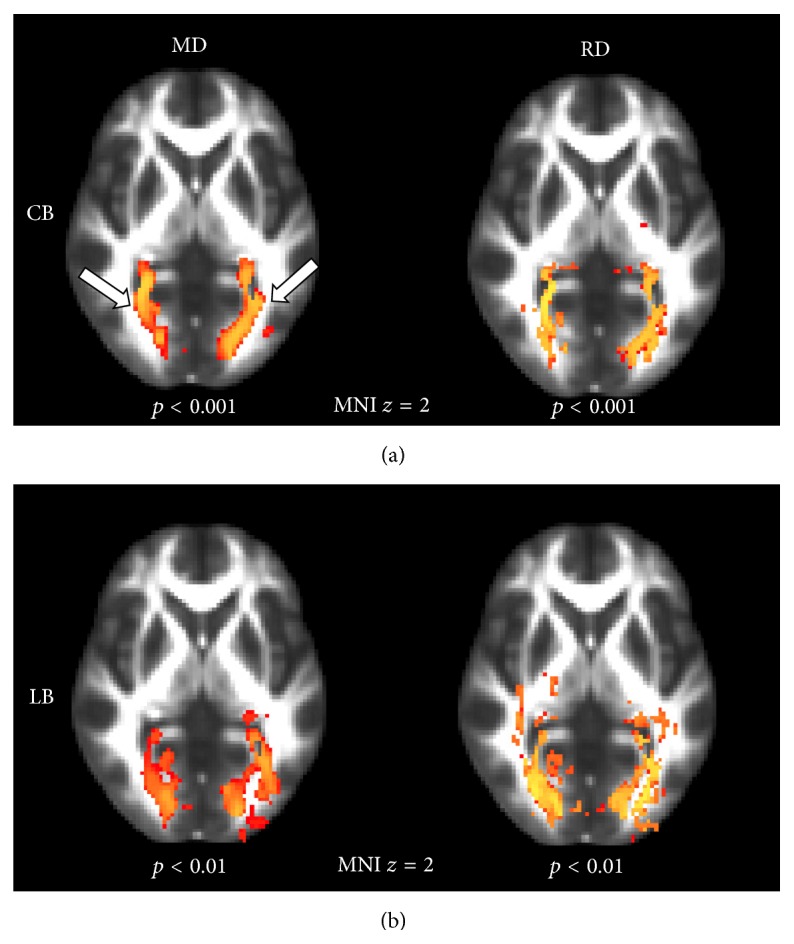
Statistical parametric MD and RD maps, showing a relative increase in regional radial diffusivity (RD) and mean diffusivity (MD) in CB and LB individuals relative to NS (red-yellow voxels). (a) shows CB individuals > NS for MD and RD: *p* < 0.001; (b) shows LB individuals > NS for MD and RD: *p* < 0.01. The RD and MD increases are accompanying the FA decrease, here illustrated in the primary visual pathway indicated by the white arrows. *p* values and background image defined as in Figure 1.

Figure 2 shows voxel-wise whole-brain microstructural differences evidenced by a decreased FA in both CB (a) and LB (b) individuals compared to the NS group (*p* < 0.001). The between-group differences in FA were mainly located within the primary visual pathway (white arrows, Figure 2) and the ventral visual processing stream. The reduction in FA was driven by increased radial diffusivity relatively to the axial diffusivity, and increased mean diffusivity, as illustrated in Figure 3. Also here, no significant difference was found in FA between PT and NS individuals, but compared with CB individuals, the PT individuals showed higher FA-values (*p* < 0.001). This confirms that the effects are related to blindness and not prematurity.

### 3.2. Tract-Specific Statistics

Differences in anatomical connectivity and microstructure between CB, LB, and NS individuals were investigated within tract-specific ROIs obtained from JHU-ICBM-DTI-81 atlas, namely, the corticospinal tract, corpus callosum, optic radiations, and ventral and dorsal visual streams.

#### 3.2.1. Regional Decreases in Anatomical Connectivity in Congenitally Blind Individuals

**Figure 4:**
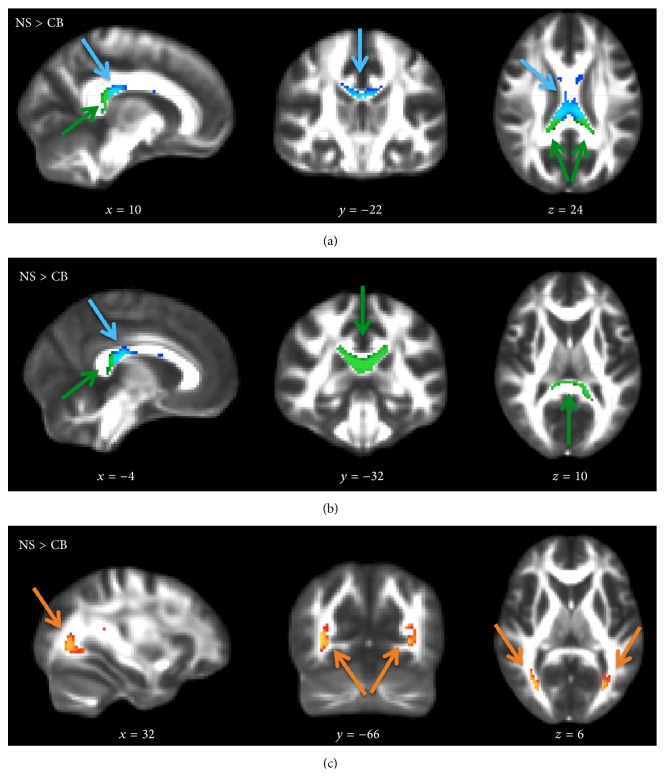
Tract ROI statistically significant differences in ACM for NS > CB individuals, voxel-based *p*_FWE_ < 0.05. Splenium (green arrow) and midbody (light blue arrow) of the corpus callosum (a, b) and the tendency of decreased ACM in the optic radiations (*p*_uncorrected_ < 0.005, orange arrow) (c). Voxels surviving the statistical threshold are overlaid on the FMRIB58_FA template. No voxels survived the threshold for NS > LB individuals. MNI-coordinates are reported in mm for *x*-, *y*-, and *z*-views.

The results of the tract-specific statistics of the ACM are shown in Table 1. Anatomical connectivity was primarily decreased in the splenium and midbody of corpus callosum in CB individuals compared to the NS group (Figure 4). No between-group differences were found in the dorsal and ventral streams or the corticospinal tract. No significant differences in anatomical connectivity were found between the LB and NS group.

#### 3.2.2. Regional Decreases in FA Associated with Blindness

**Figure 5:**
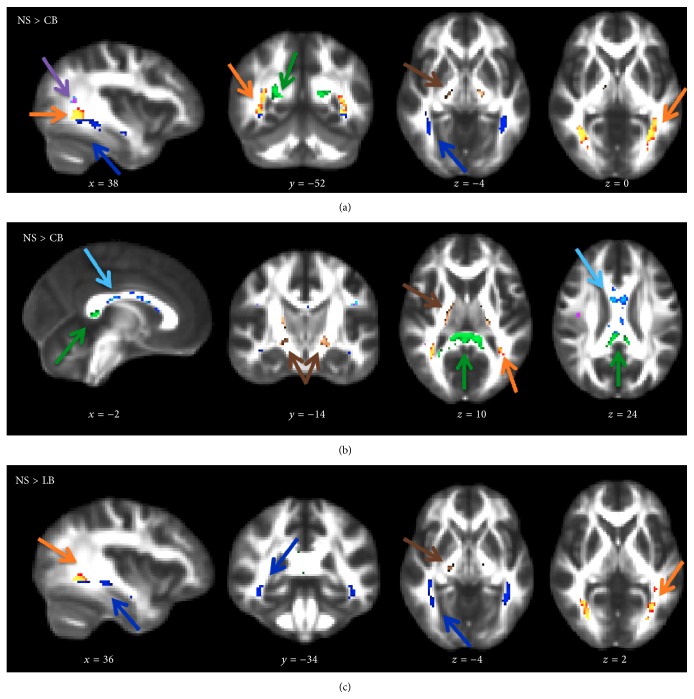
Tract ROI statistically significant differences in FA for NS > CB individuals (a, b) and NS > LB individuals (c), voxel-based *p*_FWE_ < 0.05. Optic radiations (orange arrow), ventral stream (dark blue arrow), right dorsal stream (purple arrow), splenium (green arrow), midbody (light blue arrow), and corticospinal tract (brown arrow). Voxels surviving the statistical threshold are overlaid on the FMRIB58_FA template. MNI-coordinates are reported in mm for *x*-, *y*-, and *z*-views.

Table 2 and Figure 5 illustrate the WM regions with significantly decreased FA in the CB and LB group compared to the NS group. The largest FA decreases were found in the optic radiations and the ventral stream. The right dorsal visual processing stream showed few voxels with decreased FA-values, yet the MNI-coordinates revealed overlap with the WM of the optic radiation. While the splenium of corpus callosum showed clearly reduced regional FA in CB individuals, FA-values were normal in LB subjects. Also the corticospinal tract showed a bilateral decrease in FA, primarily in the CB group.

#### 3.2.3. Congruency of Regional Differences in Connectivity and Regional Microstructure

The tractography-derived measures of anatomical connectivity and microstructure associated with blindness showed only limited spatial overlap. Although anatomical connectivity and FA differences coincided in some tract ROI, for example, the splenium, ACM differences between CB and NS subjects were more pronounced in the midbody of corpus callosum, where FA reductions were less evident. Conversely, large effects in FA were seen in the optic radiations and ventral stream, where little or no effects were highlighted with ACM. This suggests that the two measures complement each other and that ACM captures changes in brain connectivity along the tract-systems that are spatially distinct from regional changes in microstructure as reflected by FA.

### 3.3. Effect of the Intracranial Tissue Volume (ICV)

No significant difference was found in the ICV between CB and NS individuals (*p* = 0.06), whereas LB individuals showed a significantly decreased ICV (*p* = 0.01). Linear correlation was used to assess whether ACM depended linearly on the ICV for the specific ROIs and thereby could drive the differences in anatomical connectivity between CB and NS individuals. The correlation between ICV and ACM was restricted to the tract ROIs of splenium and midbody of corpus callosum, being the tract-systems where the largest ACM differences were found between CB and NS individuals. No significant correlation was found for either NS or CB individuals in the splenium ROI (NS: *r* = −0.5, *p* = 0.1; CB: *r* = −0.4, *p* = 0.2) or midbody (NS: *r* = −0.4, *p* = 0.2; CB: *r* = −0.3, *p* = 0.4). This means that the ICV is not the driving factor for the decreased ACM in CB individuals compared to NS. This is also supported by the finding of no significant differences in anatomical connectivity in the LB individuals, even though the ICV differ between the LB and NS individuals.

## 4. Discussion

Our results demonstrate that by combining ACM, expressing global connectivity, and FA, expressing local microstructure, we gain additional contrast on structural brain plasticity of the blind brain. We find that regional differences of ACM and FA in specific WM pathways depend differently on a blindness onset. We show here that anatomical connectivity is widely decreased in the callosal fibres of the splenium and midbody of corpus callosum in the congenitally blind only. In contrast, both the congenitally and late blind showed reductions in FA mainly in the optic radiations and bilaterally in the ventral stream. Thus loss of vision at an early stage of life prior to visual experience gives rise to decreased anatomical connectivity of the callosal fibres, but we found no evidence of increased FA or increased ACM as a marker of compensatory plasticity in either CB or LB individuals. ACM and DTI do not directly reflect the biological features of the tracts but help gain additional contrast on brain plastic processes. Similar to other studies in human blind subjects, the present results are based on moderately large sample sizes due to difficulties in recruiting homogenous groups of blind subjects. The results should preferably be confirmed in an independent larger population sample.

### 4.1. Decreased ACM in Corpus Callosum in CB Individuals

We find significantly decreased connectedness with ACM in subregions of the corpus callosum, that is, the splenium, and extending into the midbody in CB compared to NS individuals. The splenium mainly connects visual cortical areas through the forceps major, whereas the midbody connects both sensory and motor related cortical areas [55]. The corpus callosum has an initial exuberance in the connectivity connecting the two hemispheres. During development with normal sensory input, a phase of axonal pruning is necessary for the maturation of the callosal connections [56]. Innocenti and Frost [57] showed in a study on bilaterally enucleated kittens a 50% loss of callosal neurons projecting to visual Brodmann areas 17 and 18, caused by the lack of postnatal visual experience. The same effect could not be established after just a short period of postnatal visual stimulation [58]. This supports our results of decreased callosal ACM in CB but not LB individuals and emphasises the presence of a critical period of development in humans also. Decreased ACM in the splenium is also supported by decreased FA in the CB individuals, which is not seen in LB individuals. Decreased FA in the splenium was also reported by Yu et al. [25] in early blind subjects, whereas Bridge and Bock [26, 59] reported no differences in congenital and early blindness. We suggest that the observed combined regional ACM and DTI WM differences in the splenium could be an underlying factor of previously reported decreased functional [60] and structural [29] interhemispheric network connectivity of the occipital cortex.

Interestingly, also the midbody showed decreased ACM; however decreased FA did not support this region to the same extent as in the splenium. Thus the decreased ACM in the midbody might be related to more widespread structural connectivity changes than those related to the visual cortex. We did not expect a direct relationship between measures of FA and ACM. Although FA and ACM are both voxel-wise measures, ACM supplies tract-specific information by seeding tractography streamlines in every voxel within a tract [47]. In a study on multiple sclerosis, Lyksborg et al. [36] showed that ACM accumulated effects along the fibre pathways that were not detectable with FA. Such effects might explain the observed differences in the WM fibres within the midbody. DTI on the other hand is a very sensitive measure to local changes in microstructure. We have previously shown a general decreased GM and WM volume in the early blind [18], based on conventional structural T1-weighted MRI, which might now be explained by the observed decreased ACM and FA in the large interhemispheric callosal fibre bundle. Also Leporé et al. [61] reported T1-based volume decreases in both splenium and isthmus of corpus callosum and a tendency of increased volume of the genu of corpus callosum. More recently, Tomaiuolo et al. [62] measured the surface of callosal subregions in a large group of congenitally blind individuals and confirmed a significant decrease in the surface of the splenium whereas the isthmus and the posterior part of the midbody showed increases. Only Bock et al. [59] reported no differences between early blind, anophthalmic, and sighted subjects in the T1-weighted volume measure of the splenium as well as overall volume of the corpus callosum. As also discussed in [59], these discrepancies in the effects within the splenium of corpus callosum might be due to differences in the group analysis methodology. We used a group comparison of ACM and FA based on voxel-based analyses and atlas-based predefined ROIs of the splenium, midbody, and genu. Our ROI of the splenium might therefore include a larger area of the corpus callosum than if it had been defined individually in each subject. This explanation seems however unlikely, as also [25] reported similar decreased FA in CB individuals within individually defined splenium ROI.

If ACM is decreased in the splenium and midbody of corpus callosum, one might expect the genu to show increased ACM. For example, training-induced plasticity should have an effect on auditory and somatosensory connectivity, since CB subjects depend more on the two senses, although recent studies have indicated also superior smell abilities in congenital blind subjects [6, 15]. We do not find any increase in the ACM in the genu and also no evidence of increased FA as a marker of compensatory plasticity in either CB or LB individuals. However, increased functionality is not necessarily coupled to increased ACM or increased FA, since these measures do not directly reflect the conduction speed of the axonal bundles, coupled to axonal length and diameter [63]. During development, the brain connectivity becomes specialised by pruning of the axons, optimising the functionality. Such similar structural mechanisms might also support cross-modal plasticity.

### 4.2. Decreased Microstructural Features in CB and LB Individuals

DTI is sensitive to the local microstructural tissue environment, and we find that mainly the primary visual pathway is largely affected by visual loss, expressed as decreased FA driven by increased RD, in CB and LB individuals compared to NS. Our findings of widespread microstructural WM differences of the primary visual system are in concordance with previous reports in both animals [64] and humans [23, 24, 26]. We find that FA is decreased in both CB and LB individuals, which suggests that these differences might reflect degenerative effects in the primary visual pathways related to the onset of a visual loss. Our results also supported previous findings from our group of decreased FA bilaterally in the ventral stream that projects to higher order visual areas from the visual cortex [37]. Although we found no differences in FA in the dorsal stream, some rs-fMRI studies report a decreased functional connectivity for both streams [60]. However, changes in WM structure do not need to be directly linked with function as measured by rs-fMRI. Also, several groups have shown that blind individuals can perform motion or shape discrimination tasks by recruiting the dorsal and ventral visual pathways [12, 65–70]. Interestingly, only CB individuals showed decreased FA in the corpus callosum compared to NS, mainly in the splenium connecting the two occipital lobes. Few studies have examined specific structural differences related to blindness in nonvisual pathways, for example, the corticospinal tract. Yu et al. [25] reported an increased FA in the corticospinal tract of early blind men. We were not able to replicate this finding of increased FA in the corticospinal tract but rather found a discrete region of the tract with a decreased FA in CB individuals.

### 4.3. Combining Tract-Specific ACM, DTI Measures, and Network Analyses

We here used the two measures of ACM, expressing brain connectedness, and DTI indices, expressing regional WM microstructural features, which both provide information on specific tract-systems of interest but capture two different effects, however, indirectly coupled [36]. ACM is a new measure that gives insight into voxel-wise connectedness for a voxel within a related pathway, providing supplementary information to DTI and structural network analyses. DTI is very sensitive to voxel-wise difference in microstructural features, which at a group level need to be clustered to show a significant effect. But DTI is also heavily affected by macrostructural effects such as fibre crossing, dispersion, and undulating fibres [71–73] which means that differences in FA might be driven not only by microstructure but also by differences in axonal projections or simply by partial volume contamination from macroscopic effects and volume of the structure of interest [74].

Structural network analyses give information about whether two or more cortical regions are connected through WM fibre tracts. Thus information is obtained about whether a connection exists or not and how probable this connection is. In comparison, ACM measures the local connectivity effects along a tract-system and can thereby provide insight into the mechanisms behind the structural networks. A general decreased degree of connectivity with occipital and frontal areas was found in a structural network analysis by Shu et al. [28]. The same group later reported that the decreased degree of connectivity in the structural network of the occipital cortex was only found in congenital and early blindness but not late-onset blindness [29]. This agrees with our finding that tract-specific differences in ACM are confined to CB individuals. The observed decreased FA in the corpus callosum in CB individuals only might also reflect reduced density of fibres connecting occipital and parietal areas across the two hemispheres. However, also increased connectivity has been reported in functional brain networks of visual and language-related frontal areas [32, 60, 75–77] as measured by rs-fMRI and structural networks of motor, somatosensory, and memory areas [28, 29]. In this study, we do not find increased ACM to support the previously reported increased functional connectivity. The structural and functional network studies are somewhat contradictory, and challenges exist in comparing the different methodologies. In perspective, the understanding of the mechanisms relating functional and structural connectivity in the blind brain would benefit from a study combining functional and structural network analyses in the same group. For example, Saygin et al. [78] showed a very reliable prediction of functional activation of the fusiform gyrus in face recognition, by the anatomical connection probability with other brain areas.

## 5. Conclusion

The present study showed how ACM can be used to give complementary information to DTI indices and connectivity-based structural network analyses about the mechanisms of the plastic processes in the brain of congenitally and late-onset blind individuals. ACM identified reduced anatomical connectivity in the tract-systems of mainly the splenium and midbody of corpus callosum in congenitally blind but not late-onset blind individuals, compared to normal sighted controls. Regional voxel-based DTI revealed lower FA in the primary visual and the ventral visual stream regardless of the blindness onset, but also the splenium of corpus callosum in the congenitally blind. No region with increased anatomical connectivity or higher FA-values was observed in any of the two blind groups. This study highlights how the two different voxel-wise measures of tract-based anatomical connectivity and regional microstructure provide different information on the nature of the alterations produced by visual deprivation. They clearly provide complementary data that support a generalized model of plastic mechanisms that depend upon the onset of blindness. A future combination of all three methods of ACM, DTI, and connectivity-based structural network analyses in the same blind subject group based on the same DWI acquisition would be able to shed further light on the structural plasticity of the blind brain.

## Figures and Tables

**Table 1:** Regions of significantly decreased anatomical connectivity (ACM) in CB compared to NS individuals.

ACM	*N*_voxels_	*p*_FWE-peak_	MNI-coordinates
Tract ROI			(*x*, *y*, *z*)—mm
OR *R*	29	0.002 (uncorr.)	(36, −64, 2)
OR *L*	11	0.003 (uncorr.)	(−28, −68, 4)
ILF & IFOF *L*/*R*	—	—	—
SLF *L*/*R*	—	—	—
Splenium	24	0.02	(−12, −34, 20)
	18	0.02	(±14, −36, 10)
Midbody	95	0.02	(2, −16, 24)
Genu	—	—	—
CST *L*/*R*	—	—	—

Tract ROI defines the white matter region of interest: optic radiation (OR), inferior longitudinal fasciculus (ILF) and inferior frontooccipital fasciculus (IFOF), superior longitudinal fasciculus (SLF), and corticospinal tract (CST).

*N*_voxels_ defines the number of voxels surviving the threshold of 0.05. *p*_FWE-peak_ refers to the *p* value for the voxel with the largest *t*-test within a region. Uncorrected *p* values are given for the OR. MNI-coordinates refer to the coordinates of that voxel in mm. *L*/*R* refers to the left and right hemisphere.

**Table 2:** Regions of significantly decreased fractional anisotropy (FA) in CB and LB compared to NS individuals.

FA Tract ROI	CB			LB		
	*N*_voxels_	*p*_FWE-peak_	MNI-coordinates	*N*_voxels_	*p*_FWE-peak_	MNI-coordinates
			(*x*, *y*, *z*)—mm			(*x*, *y*, *z*)—mm
OR *L*/*R*	151	<0.0005	(−32, −52, −2)	74	<0.0005	(−34, −48, −2)
	141	<0.0005	(38, −56, −2)	86	<0.0005	(30, −72, 2)
ILF & IFOF *L*/*R*	112	<0.0005	(−38, −42, −8)	103	<0.0005	(−38, −44, −6)
	79	0.001	(36, −44, −4)	99	<0.0005	(44, −24, −14)
SLF *L*/*R*	8	0.005	(−42, −18, 26)	—	—	—
	17	0.001	(36, −54, 16)	4	0.02	(40, −14, 24)
Splenium	303	0.001	(2, −34, 8)	4	0.04	(0, −34, 10)
Midbody	46	0.004	(6, 4, 12)	1	0.05	(−4, 16, 22)
Genu	25	0.006	(−6, 20, 12)	2	0.05	(−12, 36, 8)
CST *L*/*R*	46	0.001	(−20, −16, −10)	9	0.03	(−14, −14, −6)
	46	0.001	(18, −14, 10)	15	0.03	(18, −18, −4)

Abbreviations identical to Table 1 but based on tract ROI differences in FA.

## Abbreviations

ACM: Anatomical connectivity mapping
DTI: Diffusion tensor imaging
FA: Fractional anisotropy
CB: Congenitally blind
LB: Late blind
NS: Normal sighted control
WM: White matter
GM: Grey matter.

## References

[1] Bavelier D., Neville H. J. (2002). Cross-modal plasticity: where and how?. *Nature Reviews Neuroscience*.

[2] Renier L., De Volder A. G., Rauschecker J. P. (2014). Cortical plasticity and preserved function in early blindness. *Neuroscience and Biobehavioral Reviews*.

[3] Desgent S., Ptito M. (2012). Cortical GABAergic interneurons in cross-modal plasticity following early blindness. *Neural Plasticity*.

[4] Merabet L. B., Pascual-Leone A. (2010). Neural reorganization following sensory loss: the opportunity of change. *Nature Reviews Neuroscience*.

[5] Voss P., Pike B. G., Zatorre R. J. (2014). Evidence for both compensatory plastic and disuse atrophy-related neuroanatomical changes in the blind. *Brain*.

[6] Kupers R., Ptito M. (2014). Compensatory plasticity and cross-modal reorganization following early visual deprivation. *Neuroscience and Biobehavioral Reviews*.

[7] Bedny M., Pascual-Leone A., Dodell-Feder D., Fedorenko E., Saxe R. (2011). Language processing in the occipital cortex of congenitally blind adults. *Proceedings of the National Academy of Sciences of the United States of America*.

[8] Collignon O., Vandewalle G., Voss P. (2011). Functional specialization for auditory-spatial processing in the occipital cortex of congenitally blind humans. *Proceedings of the National Academy of Sciences of the United States of America*.

[9] Watkins K. E., Shakespeare T. J., O'Donoghue M. Clare M. C. (2013). Early auditory processing in area V5/MT+ of the congenitally blind brain. *Journal of Neuroscience*.

[10] Hötting K., Röder B. (2009). Auditory and auditory-tactile processing in congenitally blind humans. *Hearing Research*.

[11] Gagnon L., Schneider F. C., Siebner H. R., Paulson O. B., Kupers R., Ptito M. (2012). Activation of the hippocampal complex during tactile maze solving in congenitally blind subjects. *Neuropsychologia*.

[12] Matteau I., Kupers R., Ricciardi E., Pietrini P., Ptito M. (2010). Beyond visual, aural and haptic movement perception: hMT+ is activated by electrotactile motion stimulation of the tongue in sighted and in congenitally blind individuals. *Brain Research Bulletin*.

[13] Sadato N., Pascual-Leone A., Grafman J. (1996). Activation of the primary visual cortex by braille reading in blind subjects. *Nature*.

[14] Théoret H., Merabet L., Pascual-Leone A. (2004). Behavioral and neuroplastic changes in the blind: evidence for functionally relevant cross-modal interactions. *Journal of Physiology Paris*.

[15] Kupers R., Beaulieu-Lefebvre M., Schneider F. C. (2011). Neural correlates of olfactory processing in congenital blindness. *Neuropsychologia*.

[16] Kupers R., Ptito M. (2011). Insights from darkness: what the study of blindness has taught us about brain structure and function. *Progress in Brain Research*.

[17] Noppeney U., Friston K. J., Ashburner J., Frackowiak R., Price C. J. (2005). Early visual deprivation induces structural plasticity in gray and white matter. *Current Biology*.

[18] Ptito M., Schneider F. C. G., Paulson O. B., Kupers R. (2008). Alterations of the visual pathways in congenital blindness. *Experimental Brain Research*.

[19] Beaulieu C. (2002). The basis of anisotropic water diffusion in the nervous system—a technical review. *NMR in Biomedicine*.

[20] Basser P. J., Mattiello J., LeBihan D. (1994). MR diffusion tensor spectroscopy and imaging. *Biophysical Journal*.

[21] Pierpaoli C., Jezzard P., Basser P. J., Barnett A., Di Chiro G. (1996). Diffusion tensor MR imaging of the human brain. *Radiology*.

[22] Le Bihan D., Mangin J.-F., Poupon C. (2001). Diffusion tensor imaging: concepts and applications. *Journal of Magnetic Resonance Imaging*.

[23] Shimony J. S., Burton H., Epstein A. A., McLaren D. G., Sun S. W., Snyder A. Z. (2006). Diffusion tensor imaging reveals white matter reorganization in early blind humans. *Cerebral Cortex*.

[24] Wang D., Qin W., Liu Y., Zhang Y., Jiang T., Yu C. (2013). Altered white matter integrity in the congenital and late blind people. *Neural Plasticity*.

[25] Yu C., Shu N., Li J., Qin W., Jiang T., Li K. (2007). Plasticity of the corticospinal tract in early blindness revealed by quantitative analysis of fractional anisotropy based on diffusion tensor tractography. *NeuroImage*.

[26] Bridge H., Cowey A., Ragge N., Watkins K. (2009). Imaging studies in congenital anophthalmia reveal preservation of brain architecture in ‘visual’ cortex. *Brain*.

[27] Shu N., Li J., Li K., Yu C., Jiang T. (2009). Abnormal diffusion of cerebral white matter in early blindness. *Human Brain Mapping*.

[28] Shu N., Liu Y., Li J., Li Y., Yu C., Jiang T. (2009). Altered anatomical network in early blindness revealed by diffusion tensor tractography. *PLoS ONE*.

[29] Li J., Liu Y., Qin W. (2013). Age of onset of blindness affects brain anatomical networks constructed using diffusion tensor tractography. *Cerebral Cortex*.

[30] Qin W., Liu Y., Jiang T., Yu C. (2013). The development of visual areas depends differently on visual experience. *PLoS ONE*.

[31] Burton H., Snyder A. Z., Conturo T. E., Akbudak E., Ollinger J. M., Raichle M. E. (2001). Adaptive changes in early and late blind: a fMRI study of Braille reading. *Journal of Neurophysiology*.

[32] Burton H., Snyder A. Z., Raichle M. E. (2014). Resting state functional connectivity in early blind humans. *Frontiers in Systems Neuroscience*.

[33] Bedny M., Konkle T., Pelphrey K., Saxe R., Pascual-Leone A. (2010). Sensitive period for a multimodal response in human visual motion area MT/MST. *Current Biology*.

[34] Bozzali M., Parker G. J. M., Serra L. (2011). Anatomical connectivity mapping: a new tool to assess brain disconnection in Alzheimer's disease. *NeuroImage*.

[35] Cercignani M., Embleton K., Parker G. J. M., Bozzali M. (2012). Group-averaged anatomical connectivity mapping for improved human white matter pathway visualisation. *NMR in Biomedicine*.

[36] Lyksborg M., Siebner H. R., Sørensen P. S. (2014). Secondary progressive and relapsing remitting multiple sclerosis leads to motor-related decreased anatomical connectivity. *PLoS ONE*.

[37] Reislev N. L., Kupers R., Siebner H. R., Ptito M., Dyrby T. B. (2015). Blindness alters the microstructure of the ventral but not the dorsal visual stream. *Brain Structure and Function*.

[38] Reese T. G., Heid O., Weisskoff R. M., Wedeen V. J. (2003). Reduction of eddy-current-induced distortion in diffusion MRI using a twice-refocused spin echo. *Magnetic Resonance in Medicine*.

[39] Andersson J. L. R., Skare S., Ashburner J. (2003). How to correct susceptibility distortions in spin-echo echo-planar images: application to diffusion tensor imaging. *NeuroImage*.

[40] Smith S. M., Jenkinson M., Woolrich M. W. (2004). Advances in functional and structural MR image analysis and implementation as FSL. *NeuroImage*.

[41] Alexander D. C., Pierpaoli C., Basser P. J., Gee J. C. (2001). Spatial transformations of diffusion tensor magnetic resonance images. *IEEE Transactions on Medical Imaging*.

[42] Jovicich J., Czanner S., Greve D. (2006). Reliability in multi-site structural MRI studies: effects of gradient non-linearity correction on phantom and human data. *NeuroImage*.

[43] Jones D. K., Basser P. J. (2004). ‘Squashing peanuts and smashing pumpkins’: how noise distorts diffusion-weighted MR data. *Magnetic Resonance in Medicine*.

[44] Cook P. A., Bai Y., Seunarine K. K., Hall M. G., Parker G. J., Alexander D. C. Camino: open-source diffusion-MRI reconstruction and processing.

[45] Behrens T. E. J., Woolrich M. W., Jenkinson M. (2003). Characterization and propagation of uncertainty in diffusion-weighted MR imaging. *Magnetic Resonance in Medicine*.

[46] Behrens T. E. J., Berg H. J., Jbabdi S., Rushworth M. F. S., Woolrich M. W. (2007). Probabilistic diffusion tractography with multiple fibre orientations: what can we gain?. *NeuroImage*.

[47] Liptrot M. G., Sidaros K., Dyrby T. B. (2014). Addressing the path-length-dependency confound in white matter tract segmentation. *PLoS ONE*.

[48] Jenkinson M., Smith S. (2001). A global optimisation method for robust affine registration of brain images. *Medical Image Analysis*.

[49] Jenkinson M., Bannister P., Brady M., Smith S. (2002). Improved optimization for the robust and accurate linear registration and motion correction of brain images. *NeuroImage*.

[50] Andersson J. L. R., Jenkinson M., Smith S. (2007). Non-linear registration aka spatial normalisation.

[51] Lilliefors H. W. (1967). On the Kolmogorov-Smirnov test for normality with mean and variance unknown. *Journal of the American Statistical Association*.

[52] Smith S. M., Nichols T. E. (2009). Threshold-free cluster enhancement: addressing problems of smoothing, threshold dependence and localisation in cluster inference. *NeuroImage*.

[53] Winkler A. M., Ridgway G. R., Webster M. A., Smith S. M., Nichols T. E. (2014). Permutation inference for the general linear model. *NeuroImage*.

[54] Mori S., Wakana S., Nagae-Poetscher L. M., van Zijl P. C. M. (2005). *MRI Atlas of Human White Matter*.

[55] Catani M., Thiebaut de Schotten M. (2012). *Atlas of Human Brain Connections*.

[56] Innocenti G. M., Price D. J. (2005). Exuberance in the development of cortical networks. *Nature Reviews Neuroscience*.

[57] Innocenti G. M., Frost D. O. (1980). The postnatal development of visual callosal connections in the absence of visual experience or of the eyes. *Experimental Brain Research*.

[58] Innocenti G. M., Frost D. O., Illes J. (1985). Maturation of visual callosal connections in visually deprived kittens: a challenging critical period. *Journal of Neuroscience*.

[59] Bock A. S., Saenz M., Tungaraza R., Boynton G. M., Bridge H., Fine I. (2013). Visual callosal topography in the absence of retinal input. *NeuroImage*.

[60] Liu Y., Yu C., Liang M. (2007). Whole brain functional connectivity in the early blind. *Brain*.

[61] Leporé N., Voss P., Lepore F. (2010). Brain structure changes visualized in early- and late-onset blind subjects. *NeuroImage*.

[62] Tomaiuolo F., Campana S., Collins D. L. (2014). Morphometric changes of the corpus callosum in congenital blindness. *PLoS ONE*.

[63] Caminiti R., Carducci F., Piervincenzi C. (2013). Diameter, length, speed, and conduction delay of callosal axons in macaque monkeys and humans: comparing data from histology and magnetic resonance imaging diffusion tractography. *Journal of Neuroscience*.

[64] Chan K. C., Cheng J. S., Fan S., Zhou I. Y., Yang J., Wu E. X. (2012). In vivo evaluation of retinal and callosal projections in early postnatal development and plasticity using manganese-enhanced mri and diffusion tensor imaging. *NeuroImage*.

[65] Amedi A., Malach R., Hendler T., Peled S., Zohary E. (2001). Visuo-haptic object-related activation in the ventral visual pathway. *Nature Neuroscience*.

[66] Pietrini P., Furey M. L., Ricciardi E. (2004). Beyond sensory images: object-based representation in the human ventral pathway. *Proceedings of the National Academy of Sciences of the United States of America*.

[67] Poirier C., Collignon O., Scheiber C. (2006). Auditory motion perception activates visual motion areas in early blind subjects. *NeuroImage*.

[68] Ptito M., Matteau I., Gjedde A., Kupers R. (2009). Recruitment of the middle temporal area by tactile motion in congenital blindness. *NeuroReport*.

[69] Renier L. A., Anurova I., De Volder A. G., Carlson S., VanMeter J., Rauschecker J. P. (2009). Multisensory integration of sounds and vibrotactile stimuli in processing streams for ‘what’ and ‘where’. *The Journal of Neuroscience*.

[70] Collignon O., Dormal G., Albouy G. (2013). Impact of blindness onset on the functional organization and the connectivity of the occipital cortex. *Brain*.

[71] Lasič S., Szczepankiewicz F., Eriksson S., Nilsson M., Topgaard D. (2014). Microanisotropy imaging: quantification of microscopic diffusion anisotropy and orientational order parameter by diffusion MRI with magic-angle spinning of the q-vector. *Frontiers in Physics*.

[72] Szczepankiewicz F., Lasič S., van Westen D. (2015). Quantification of microscopic diffusion anisotropy disentangles effects of orientation dispersion from microstructure: applications in healthy volunteers and in brain tumors. *NeuroImage*.

[73] Jespersen S. N., Lundell H., Sønderby C. K., Dyrby T. B. (2014). Commentary on ‘microanisotropy imaging: quantification of microscopic diffusion anisotropy and orientation of order parameter by diffusion MRI with magic-angle spinning of the q-vector’. *Frontiers of Physics*.

[74] Vos S. B., Jones D. K., Viergever M. A., Leemans A. (2011). Partial volume effect as a hidden covariate in DTI analyses. *NeuroImage*.

[75] Wang D., Qin W., Liu Y., Zhang Y., Jiang T., Yu C. (2014). Altered resting-state network connectivity in congenital blind. *Human Brain Mapping*.

[76] Watkins K. E., Cowey A., Alexander I. (2012). Language networks in anophthalmia: maintained hierarchy of processing in ‘visual’ cortex. *Brain*.

[77] Heine L., Bahri M. A., Cavaliere C. (2015). Prevalence of increases in functional connectivity in visual, somatosensory and language areas in congenital blindness. *Frontiers in Neuroanatomy*.

[78] Saygin Z. M., Osher D. E., Koldewyn K., Reynolds G., Gabrieli J. D. E., Saxe R. R. (2012). Anatomical connectivity patterns predict face selectivity in the fusiform gyrus. *Nature Neuroscience*.

